# Psychometric validation of the Polish version of the Utrecht Gender Dysphoria Scale - Gender Spectrum questionnaire

**DOI:** 10.3389/fpsyt.2026.1793878

**Published:** 2026-04-01

**Authors:** Agata Tokarek, Aleksandra Koczut, Agata Gasiorowska

**Affiliations:** Faculty of Psychology in Wroclaw, SWPS University, Wroclaw, Poland

**Keywords:** gender dysphoria, gender identity, psychometrics, surveys and questionnaires, transgender persons

## Abstract

**Background:**

Accessible and inclusive tools for assessing gender dysphoria in Polish-speaking populations remain limited. To bridge this gap in care, there is an urgent need for accessible, psychometrically sound tools to assess Polish gender-diverse individuals. In this study, we aimed to adapt the Polish version of the Utrecht Gender Dysphoria Scale - Gender Spectrum to address a critical gap in assessment tools that include nonbinary, binary transgender, and cisgender individuals.

**Methods:**

In Study 1 (N = 1,057), the 18-item UGDS-GS was translated into Polish using a backward translation procedure. Its factorial structure, internal consistency, and construct validity were examined in an online sample of Polish adults, including binary transgender, nonbinary, and cisgender participants recruited via social media and the Prolific platform between July and August 2025. Participants completed the UGDS-GS along with measures of anxiety, depression, and gender dysphoria. In Study 2, an independent sample of Polish adults (N = 245 at T1) recruited via Prolific completed the UGDS-GS-PL. Additional confirmatory factor analyses were conducted, and stability over time was assessed using a three-week test-retest design.

**Results:**

Confirmatory factor analysis did not support the original two-factor structure. Exploratory factor analysis instead identified a three-factor solution *(Gender dysphoria*, *Puberty-related distress*, *Gender affirmation*) which was subsequently confirmed in an independent sample using confirmatory factor analysis. We found that the scale’s two distress-related dimensions capture relatively stable individual differences across the full gender spectrum. Moreover, the scale demonstrated good internal consistency and good measurement stability. ROC analyses demonstrated excellent screening accuracy for the *Gender dysphoria* subscale (AUC = .97), good accuracy for *Puberty-related distress* (AUC = .81), and limited accuracy for *Gender affirmation* (AUC = .62).

**Conclusions:**

The scale provides a valuable alternative to existing binary-focused instruments and demonstrates that gender-neutral assessment of gender dysphoria is both feasible and psychologically viable in the Polish language.

## Background

Contemporary clinical consensus conceptualizes gender diversity as a natural variation of human experience rather than a pathology (1). This evolving understanding is reflected in modern diagnostic frameworks: the ICD-11 uses the term Gender Incongruence to describe a consistent mismatch between one’s experienced and assigned gender (2). Conversely, the DSM-5 uses the term Gender Dysphoria, which specifically emphasizes the clinically significant distress that may arise from such incongruence (3). However, despite robust evidence linking gender dysphoria to diminished well-being and mental health outcomes (4, 5), the condition is frequently overlooked in clinical practice. Such oversight significantly delays access to essential Gender Affirming Care (GAC), unnecessarily prolonging patient distress (6). Consequently, to bridge this gap in care, there is a critical need for accessible, psychometrically sound tools to evaluate gender dysphoria within the Polish healthcare context.

The existing Polish gender dysphoria assessment tool, the Gender Identity/Gender Dysphoria Questionnaire for Adolescents and Adults (GIDYQ-AA-PL) (7, 8), is limited by its inherent binary conceptualization of gender Specifically, the questionnaire items are framed around internal experiences, such as feeling like, thinking about, or dreaming of oneself as either a woman or a man. This binary structure is further reinforced by the GIDYQ-AA-PL’s availability in only two grammatical forms (masculine and feminine), which significantly restricts its applicability and inclusivity for non-binary individuals. Given the increasing visibility of gender-diverse individuals in Poland — as reflected in the growing participation in recent edition of the Nonbinary Census (9) and a 7% increase in the transgender respondents in report on the social situation of LGBTQIA+ individuals (10) — and the documented limitations of the GIDYQ-AA-PL, we decided to adapt an alternative instrument: the Utrecht Gender Dysphoria Scale - Gender Spectrum (11, 12). This instrument is a revised version of the earlier Utrecht Gender Dysphoria Scale (13), specifically designed to move beyond the binary conceptualization of gender. The UGDS-GS consists of 18 items measured on a 5-point Likert scale. Crucially, the language was intentionally developed to be gender-neutral, accurately capturing the experiences of individuals across the entire gender spectrum. The scale has been successfully adapted into other languages, including Thai (14), Chinese (15), and Turkish (16). In this project, we conducted two studies with the aim of adapting this scale to Polish, testing its factorial structure, validity, and reliability, and providing cut-off points for potential screening.

### Psychometric quality of UGDS-GS

The original English version of UGDS-GS consists of two dimensions: Gender dysphoria and Gender affirmation. The Gender affirmation subscale consists of four positively valenced items indicating complete agreement with the benefits of living in the affirmed gender. In turn, Gender dysphoria is measured with 14 items that indicate distress about one’s physical characteristics, expected behaviors, and sense of self in their assigned sex (11, 12). The scale demonstrates a large degree of invariance across binary transgender, nonbinary/genderqueer, and cisgender LGBQ subgroups (12). It also demonstrated the convergent and discriminant validity in LGBTQ populations, although the Gender dysphoria dimension performed better than the Gender affirmation dimension. In the UK population, Gender dysphoria correlated with some but not all subscales from genderqueer identity (GQI) subscales (11). In the Chinese population, the Gender dysphoria subscale was positively moderately associated with GIDYQ-AA-PL scores (*r* = .36), depression (*r* = .32), and anxiety (*r* = .30), whereas the Gender affirmation subscale was weakly correlated with anxiety (*r* = .04) and negatively weakly correlated with GIDYQ-AA-PL (*r* = -.19) (15). In the Turkish adaptation (where only the Gender dysphoria subscale was retained), UGDS-GS scores showed weak to moderate correlations with several RSES and YSR subscales, including depressive affect (*r* = .20), psychosomatic symptoms (*r* = .26), anxiety/depression (*r* = .28), and social problems (*r* = .42) (16). In the Thai adaptation (14), the authors did not provide information on the convergent validity of the UGDS-GS. In all versions of the UGDS-GS, reliability of the scale was assessed through internal consistency, with Cronbach’s alphas of.96 in the Thai adaptation,.89 in the Chinese adaptation, and.94 in the Turkish adaptation.

In sum, the UGDS-GS is a well-validated, psychometrically robust measure of gender dysphoria designed to capture experiences across the full gender spectrum. It demonstrates strong internal consistency as well as evidence of convergent and discriminant validity across diverse populations. Importantly, because the UGDS-GS uses intentionally gender-neutral language and avoids binary assumptions, it overcomes key limitations of other tools such as the UGDS in its original version or GIDYQ-AA-PL. The scale has already been successfully translated into multiple languages (Thai, Chinese, Turkish) demonstrating good reliability and meaningful validity. Therefore, in this project, we aimed to adapt the UGDS-GS to the Polish context and rigorously tested for factorial structure, reliability, validity, and screening cut-offs in two preregistered studies (total *N* = 1265), providing a psychometrically sound and inclusive instrument suitable for clinical and research use in Poland.

## Study 1

The aim of this study was to provide evidence for the structure and validity of the Polish adaptation of the UGDS-GS (11, 12). This 18-item questionnaire was translated into Polish using a forward–backward translation (back-translation) procedure in line with established cross-cultural adaptation guidelines (17). First, three independent individuals (including a translator from the Neutral Language Council, a Polish queer collective dedicated to assembling, researching, shaping, and promoting gender-neutral and nonbinary language) prepared a forward translation. These versions were compared and synthesized into a consensual Polish version. Another three people (including a non-binary person) then translated the consensual version back to English. Finally, all authors reviewed the backtranslation against the original version and made necessary corrections to ensure semantic and conceptual equivalence, thereby creating the final scale. Although the Polish language distinguishes feminine and masculine grammatical forms, our focus was to develop only items that used gender-free grammar and pronouns to preserve inclusivity and conceptual consistency with the original instrument. The final version showed good readability (Gunning Fog index = 6.73), which reflects the approximate number of years of formal education required to understand the text (18), suggesting it is understandable to individuals with primary education.

In Study 1, we invited a sample of Polish participants with the following aims: (1) to test the original structure of the scale, as proposed by McGuire et al. (11, 12), (2) to verify internal consistency of the dimensions of UGDS-GS-PL, and (3) to establish the convergent and divergent validity of the subscales that make up the UGDS-GS-PL (19). In line with the preregistration, we expected that (1) the Gender dysphoria subscale would positively correlate with GIDYQ-AA-PL, and also positively but weaker with anxiety (GAD-7) and depression (CESD-R); (2) Transgender and nonbinary individuals will score higher on the Gender dysphoria subscale than cisgender LGBQ+ participants; and that (3) gender identity would moderate the relationship between the Gender dysphoria and the Gender affirmation subscales such that this association is positive and significant among trans and non-binary participants, but weaker or even non-significant among cisgender participants. We did not have specific expectations regarding the relationships between the Gender affirmation subscale and levels of depression, anxiety, or other psychological constructs. Similarly, we did not have expectations regarding differences in Gender affirmation scores between cisgender, transgender, and non-binary participants. This study’s hypotheses, design, sample size, exclusions, and analyses were preregistered at https://aspredicted.org/pch2-hcf5.pdf.

### Methods

#### Ethical considerations

This project adheres to ethical guidelines specified in the APA Code of Conduct and the Declaration of Helsinki, and was approved by the Ethics Committee for Human Research of SWPS University, Faculty of Psychology in Wroclaw. Across studies, all participants provided informed consent within the survey system. All data and preregistrations, together with the Polish version of the UGDS-GS, are available at https://researchbox.org/5006.

#### Participants

In line with our preregistration, we planned to recruit at least 500 participants from the following groups: (a) transgender, (b) nonbinary, (c) cisgender, non-heterosexual (e.g., lesbian, gay, bisexual, queer, pansexual, asexual). However, we decided to expand our recruitment to cisgender heterosexual individuals. Participants were recruited online via LGBTQ+-focused Facebook groups and the Prolific Academic platform (20). We recruited 1079 participants (age: *M* = 25.55, *SD* = 7.38): 408 non-binary (including agender), 186 binary transgender individuals, 329 non-heterosexual cisgender individuals, and 156 heteronormative cisgender individuals; 839 via Facebook and 242 via Prolific. Twenty-two of them failed to respond to one or more of our preregistered attention checks and were excluded from the analysis. The final sample consisted of 1057 participants (age: *M* = 25.55, *SD* = 7.34): 401 non-binary (including agender; age: *M* = 24.18, *SD* = 6.21), 183 binary transgender (age: *M* = 23.89, *SD* = 7.00), 319 non-heterosexual cisgender (age: *M* = 25.47, *SD* = 7.15), and 154 heteronormative cisgender (age: *M* = 31.27, *SD* = 8.10). 820 of them were recruited via Facebook and 237 via Prolific.

#### Procedure

After giving informed consent, participants provided basic demographic information, including age, sex assigned at birth, gender identity, and sexual orientation. Then, they completed the following questionnaires: (1) Utrecht Gender Dysphoria Scale - Gender Spectrum (11, 12), in our translation, with a 5-point Likert scale, (2) Generalized Anxiety Disorder 7 (21), a 7-item measure assessing generalized anxiety disorder symptoms with 4-point Likert scale (Cronbach’s α = .91); (3) Center for Epidemiologic Studies Depression Scale (22, 23), a 14-item self-report instrument measuring depressive symptoms with 5-point Likert scale (Cronbach’s α = .93), and (4) Gender Identity/Gender Dysphoria Questionnaire for Adolescents and Adults (7, 8), a 27-item questionnaire assessing gender dysphoria with 5-point Likert scale (Cronbach’s α = .96). Completion of the GIDYQ-AA-PL was not obligatory, as we anticipated that some of its items might be uncomfortable or difficult for participants to answer. The order of questionnaires 1-3 and the questions within them was randomized. The GIDYQ-AA-PL was always presented last to avoid possible influences of its content on responses to the other measures. In line with preregistration, we included two attention checks (“Please select ‘Strongly disagree’ for this item” and “Which animal is the largest?” with response options: elephant, mouse, scorpion, stork). Participation was voluntary and, for the majority of participants, no financial compensation was provided; however, in the supplementary recruitment conducted via Prolific, participants received £1.05 for their participation.

#### Statistical analyses

All statistical analyses were conducted using Jamovi 2.6.23 and R 4.4.3. Prior to analysis, participants who failed preregistered attention checks were excluded.

To examine the factorial structure of the UGDS-GS-PL, as preregistered, we first conducted confirmatory factor analyses (CFA) testing the original two-factor model and an alternative unidimensional model. When preregistered CFA models showed inadequate fit, we divided the whole sample randomly into two subsets: *n_1_* = 516, which we used for EFA, and *n_2_* = 541, which we used for second CFA. Convergent and theoretical validity were examined using Pearson correlations between UGDS-GS-PL subscales and measures of gender incongruence (GIDYQ-AA-PL), anxiety (GAD-7), and depression (CESD-R). Group differences across cisgender, nonbinary, and binary transgender participants were tested using one-way ANOVAs with Bonferroni *post hoc* comparisons. To evaluate potential screening utility, we conducted ROC analyses to assess the ability of UGDS-GS-PL subscales to distinguish cisgender from transgender/nonbinary participants. Optimal cutoffs were estimated using the *cutpointr* package in R (24) with 10,000 bootstrap iterations.

### Results

#### Structure of the scale

As preregistered, we conducted Confirmatory Factor Analysis (CFA) to evaluate the factorial structure of the scale. We tested two competing models: (1) a two-factor solution, in which we treat Gender affirmation and Gender dysphoria as correlated but distinct constructs, and (2) a one-factor solution, in which we conceptualize gender-related distress and affirmation as opposite ends of a single dimension. We examined goodness of fit using multiple indices: χ^2^/*df*, the Root Mean Square Error of Approximation (RMSEA) and its 90% confidence interval (90% CI), the Comparative Fit Index (CFI), and the Tucker-Lewis fit index (TLI). We used multiple fit indices to assess different types of model fit (e.g., model parsimony, absolute fit) and to provide a more reliable, conservative evaluation (25). The model was evaluated using the criteria recommended by Hu and Bentler (26). The lower boundary for an acceptable fit for CFI and TLI was 0.90, and the upper boundary for an acceptable fit for RMSEA and SRMR was .08. As McGuire et al. (12) did not provide information about the estimation method used in their work on the English version of the scale, we conservatively used a Maximum Likelihood with Robust Errors (MLM) estimation method (27, 28). A confirmatory factor analysis (CFA) conducted in Jamovi yielded a poor fit for this model, based on some but not all indices: χ^2^/*df* = 7.70, RMSEA = .111, 90% CI [.105, .117], SRMR = .079, CFI = .90, TLI = 0.89. Inspection of model diagnostics (including residuals and modification indices) suggested minor residual covariances between conceptually related item pairs; however, freeing these parameters resulted in only marginal improvements in fit and did not resolve the broader pattern of misfit. Given the limited impact of these localized adjustments and to avoid *post-hoc*, data-driven model modifications, we did not pursue further respecification. The fit for an alternative unidimensional CFA model that grouped all 18 items in a single factor representing gender dysphoria was only slightly better than the fit with our theoretically derived scale structure, χ^2^/*df* = 12.97, RMSEA = .106, 90% CI [.102,.111], SRMR = .071, CFI = .91, TLI = 0.90. Therefore, we decided to conduct an exploratory factor analysis (EFA) to empirically examine the underlying structure of the data without imposing strict a priori constraints. We divided the whole sample randomly into two subsets: *n_1_* = 516, which we used for EFA, and *n_2_* = 541, which we used for CFA.

**Figure 1:**
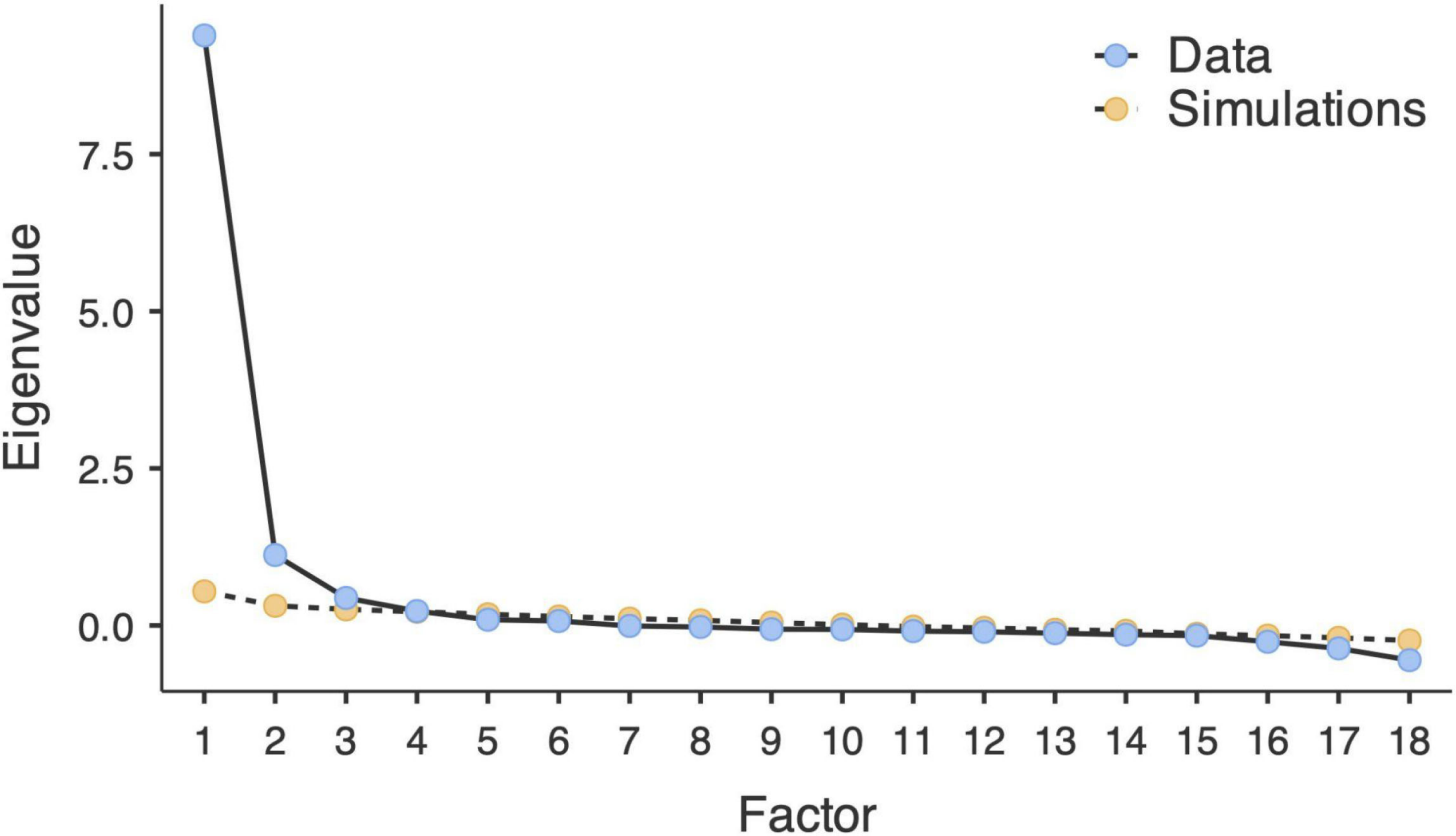
Scree plot for the UGDS-PL.

The KMO measure of sampling adequacy calculated in the first subsample was 0.96, and Barlett’s test of sphericity was significant, χ^2^(153) = 7535.35, *p* <.001, confirming that the quality of our data was sufficient to conduct EFA. Although the parallel analysis suggested four factors, the visual inspection of the scree plot identified three (see **Figure 1**). We therefore conducted an EFA with the Oblimin rotation and analyzed items with absolute factor loadings of 0.4 or higher. The three-factor model explained 62.87% of the total variance in the data, with the majority (45.28%) being explained by the Gender dysphoria factor (GD), followed by the new factor indicating Puberty-related distress (PRD, 9.58%) and Gender affirmation (GA, 8.01%). The factor loadings are presented in **Table 1**.

**Table 1:** Standardized factor loading from EFA (study 1).

Items	Factor		
	1	2	3
13 I feel hopeless if I have to stay in my assigned sex.	**.95**	-.01	.03
2 Every time someone treats me like my assigned sex I feel hurt.	**.93**	-.04	.07
17 I feel uncomfortable behaving like my assigned sex.	**.93**	-.02	-.10
6 I feel unhappy when I have to behave like my assigned sex.	**.92**	-.00	-.07
15 I feel unhappy because I have the physical characteristics of my assigned sex.	**.86**	.04	-.06
16 I hate my birth assigned sex.	**.84**	.03	.00
12 My life would be meaningless if I would have to live as my assigned sex.	**.83**	-.00	.11
5 A life in my affirmed gender is more attractive for me than a life in my assigned sex.	**.74**	.02	.06
18 It would be better not to live, than to live as my assigned sex.	**.73**	.09	.11
7 It is uncomfortable to be sexual in my assigned sex.	**.68**	.13	-.14
10 I wish I had been born as my affirmed gender.	**.45**	-.01	.16
14 I feel unhappy when someone misgenders me.	**.43**	.16	.30
8 Puberty felt like a betrayal.	.26	**.62**	.02
9 Physical sexual development was stressful.	-.08	**.87**	.01
11 The bodily functions of my assigned sex are distressing for me (i.e. erection, menstruation).	.36	**.43**	-.04
1 I prefer to behave like my affirmed gender.	.08	.03	**.58**
3 It feels good to live as my affirmed gender.	-.24	-.03	**.59**
4 I always want to be treated like my affirmed gender.	.10	.02	**.73**

Factor loadings >.40 are presented in bold.

Then, we conducted a CFA to test the three-factor model in the second subsample. The model demonstrated acceptable values for most of the fit indices, χ^2^/*df* = 7.70, RMSEA = .089, 90% CI [.083, .096], SRMR = .077, CFI = .94, TLI = 0.93. All standardized factor loadings are significant and exceed .50 (**Table 2**). Therefore, although the RMSEA fit index is suboptimal, we decided to use the three-factor structure of the UGDS-PL scale for further analyses. Accordingly, Gender dysphoria was calculated as the sum of items 2, 5, 6, 7, 10, 12, 13, 14, 15, 16, 17, and 18; Puberty-related distress as the sum of items 8, 9, and 11; and Gender affirmation as the sum of items 1, 3, and 4.

**Table 2:** Standardized factor loadings for the three-Factor Solution From CFA in Study 1 (subsample n_2_) and Study 2 (T1).

Factor	Item	Study 1 subsample n_2_	Study 2
Gender dysphoria	UGDS_GS_5	.76	.65
	UGDS_GS_2	.92	.91
	UGDS_GS_6	.92	.90
	UGDS_GS_7	.71	.72
	UGDS_GS_10	.53	.32
	UGDS_GS_12	.84	.91
	UGDS_GS_13	.94	.93
	UGDS_GS_14	.61	.44
	UGDS_GS_15	.87	.83
	UGDS_GS_16	.85	.83
	UGDS_GS_17	.89	.89
	UGDS_GS_18	.79	.82
Puberty-related distress	UGDS_GS_8	.86	.82
	UGDS_GS_9	.76	.67
	UGDS_GS_11	.70	.71
Gender affirmation	UGDS_GS_1	.93	.71
	UGDS_GS_3	.56	.40
	UGDS_GS_4	.50	.84

##### Internal consistency

Internal consistency coefficients (ω) were calculated using the full sample from Study 1, as reliability estimation does not involve model cross-validation in the same way as structural analyses. Gender dysphoria demonstrated excellent internal consistency (ω = .96, AVE = .66), with all discrimination indices also exceeding .60. Puberty-related distress (PRD) showed good internal consistency (ω = .82, AVE = .60), with all discrimination indices exceeding .60. Gender affirmation demonstrated questionable to acceptable internal consistency (ω = .68, AVE = .48). While the average variance explained for this factor this falls slightly below the commonly accepted threshold of .50, all item discrimination indices exceeded .45, indicating that each item adequately differentiates between respondents with high and low levels of Gender affirmation. This suggests the items are functioning appropriately despite the moderate overall reliability.

##### Validity

###### Intercorrelations between subscales

The separate bivariate correlation analysis revealed a distinct pattern of relationships between the three dimensions, providing insight into the scale’s internal structure. Gender affirmation showed weak correlations with both other dimensions, with *r* = .09, *p* < .01 for Puberty-related distress, and *r* = .21, *p* < .001 for Gender dysphoria. These low correlations suggest that Gender affirmation represents a relatively independent construct, capturing positive aspects of gender identity that are largely distinct from distress-related experiences. In contrast, Puberty-related distress and Gender dysphoria demonstrated a strong positive correlation (*r* = .73, *p*  <.001). This substantial relationship indicates considerable overlap between distress related to pubertal development and broader gender dysphoria. The high correlation suggests these dimensions share common variance and may reflect related aspects of gender-related distress, though they remain empirically distinguishable constructs.

###### Theoretical validity of the UGDS-GS-PL scale

In **Table 3**, we present the results of the Pearson correlation analysis, which tests the theoretical validity of the UGDS-GS-PL scale. As we hypothesized, Gender dysphoria demonstrated strong convergence with the GIDYQ-AA-PL and showed significant positive but weaker correlations with anxiety and depression. This pattern shows that gender dysphoria is associated with psychological distress, though not reducible to general psychopathology.

**Table 3:** Correlations between three dimensions of UGDS-GS-PL, gender dysphoria, general anxiety, and depression.

Dimensions of UGDS-GS-PL	Gender dysphoria (GIDYQ-AA-PL) (n = 867)	General Anxiety (GAD)	Depression (CESD-R)
Gender Dysphoria	.90***	.13 ***	.24***
Puberty-related Distress	.65***	.25***	.32***
Gender Affirmation	.20***	-.10***	-.11***

*p < .05; **p < .01; ***p < .001.

Puberty-related distress also showed strong convergent validity with the GIDYQ-AA-PL and demonstrated weak-to-moderate positive correlations with both anxiety and depression, suggesting associations with broader psychological difficulties. Because Puberty-related distress emerged as a new factor theoretically associated with age, we examined its relationship with participants’ age. We observed a negative association among cisgender participants (*r* = −.12, *p* = .012), but no such association was observed among nonbinary (*r* = .01, *p* = .766) or binary transgender participants (*r* = .02, *p* = .763).

Gender affirmation demonstrated a weak positive correlation with the GIDYQ-AA-PL. This might be considered counterintuitive, as higher Gender affirmation could theoretically correspond with lower Gender dysphoria, but it aligns with the results by McGuire et al. (2020). The negative associations with anxiety and depression suggest Gender affirmation may be protective against psychological distress, though these relationships were modest.

Further, we conducted one-way ANOVAs and *post-hoc* comparisons with Bonferroni correction to compare levels of each factor across cisgender, non-binary, and binary-transgender participants. As preregistered, we hypothesized that transgender and nonbinary individuals would score higher on the Gender dysphoria subscale than cisgender LGBQ+ participants, and we also extended this prediction to Puberty-related distress. We did not have expectations regarding differences in Gender affirmation scores between cisgender, transgender, and non-binary participants.

For Gender dysphoria dimension, we found significant and very large differences between the three groups, *F*(2, 1054) = 1845.19, *p* < .001, *η²* = .78. Cisgender participants reported significantly lower levels of Gender dysphoria compared to both nonbinary participants, *t*(1054) = −46.64, *p_Bonferroni_* < .001, *d* = −3.17, and non-binary transgender participants: *t*(1054) = −53.04, *p_Bonferroni_* < .001, *d* = −4.62. Binary transgender participants also reported significantly higher levels of Gender dysphoria than nonbinary participants, *t*(1054) = −16.27, *p_Bonferroni_* < .001, *d* = −1.45. These results confirm the theoretical validity of this dimension (see **Table 4**).

**Table 4:** Levels of Gender dysphoria, Puberty-related distress, and Gender affirmation measured by UGDS-GS-PL across cisgender, non-binary, and binary-transgender participants.

Group	Gender dysphoria *M*(*SD*)	Puberty-related distress *M*(*SD*)	Gender affirmation *M*(*SD*)
Cingender	20.29 (5.76)	6.32 (2.90)	12.19 (2.54)
Nonbinary	43.01 (8.85)	10.34 (3.12)	12.42 (2.35)
Binary Transgender	53.44 (6.34)	11.95 (2.55)	14.04 (1.58)

For Puberty-related distress dimension, we found a significant and large effect, *F*(2, 1054) = 330.81, *p* <.001, *η²* = .39. Cisgender participants reported significantly lower levels of Puberty-related Distress compared to nonbinary participants: *t*(1054) = −20.23, *p_Bonferroni_* < .001, *d* = −1.37 and binary transgender participants: *t*(1054) = −22.07, *p_Bonferroni_* < .001, *d* = −1.92. The difference between binary transgender and nonbinary participants was also significant: *t*(1054) = −6.15, *p_Bonferroni_* < .001, *d* = −0.55 (see **Table 4**).

The differences between the three groups in Gender affirmation were significant, but weaker than for the two other dimensions, *F*(2, 1054) = 31.73, *p* < .001, *η^2^* = .08. We did not find a significant difference between cisgender and nonbinary participants, *t*(1054) = −1.46, *p_Bonferroni_* = .431, *d* = -0.10. Binary transgender participants scored significantly higher on Gender affirmation compared to both cisgender participants, *t*(1054) = −9.15, *p_Bonferroni_* < .001, *d* = -0.80, and non-binary participants: *t(*1054*)* = −7.82, *p_Bonferroni_* < .001, *d* = -0.70 (see **Table 4**).

In sum, the preregistered two-factor structure of the UGDS-GS-PL was not supported, as this model showed inadequate fit, and the alternative one-factor model performed only slightly better. Instead, our exploratory factor analysis revealed a clear three-factor solution: Gender dysphoria, Puberty-related distress, and Gender affirmation, which we subsequently confirmed in a separate CFA with acceptable fit and strong item loadings, providing evidence for the scale’s factorial validity. We also found excellent internal consistency for Gender dysphoria, good consistency for Puberty-related distress, and moderate but acceptable reliability for Gender affirmation, supported by solid item discrimination. The pattern of intercorrelations further supported the internal structure: the two distress-related dimensions were strongly related, while Gender affirmation showed only weak associations with them, indicating that it represents a distinct, largely protective construct. We additionally confirmed convergent and theoretical validity, as the distress-related dimensions correlated strongly with established measures of gender dysphoria and modestly with anxiety and depression, whereas Gender affirmation showed weak positive associations with dysphoria and small negative associations with psychological distress. Finally, we found the expected group differences, with transgender and nonbinary participants scoring substantially higher on distress-related dimensions than cisgender participants, providing further support for the theoretical validity of the UGDS-GS-PL.

#### Auxiliary analyses

**Figure 2:**
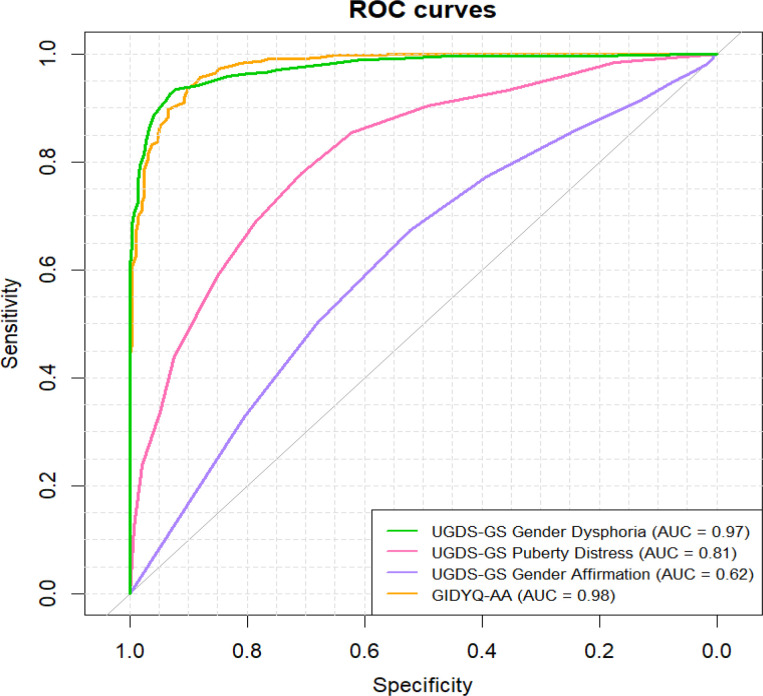
ROC curves for UGDS-GS-PL gender dysphoria, UGDS-GS-PL Puberty-related distress, UGDS-GS-PL Gender affirmation, and GIDYQ-AA-PL.

To establish screening cutoffs for the UGDS-GS subscales that could support a preliminary assessment of gender dysphoria, we aimed to evaluate how well these subscales distinguish transgender and nonbinary participants from cisgender participants. Because the GIDYQ-AA-PL is currently the only psychometric tool for screening for gender dysphoria in Poland, we sought to directly compare the classification performance of our new scale with that of this established measure. To do so, we conducted ROC analyses to assess the ability of the UGDS-GS subscales and the GIDYQ-AA-PL to differentiate participants by gender identity (cisgender vs. transgender/nonbinary). These analyses were performed on Study 1 participants who completed both the UGDS-GS-PL and the GIDYQ-AA-PL (age: *M* = 24.22, *SD* = 6.69), consisting of 293 cisgender, 395 non-binary, and 179 binary transgender participants. We employed the method that maximizes accuracy using the *cutpointr* package in *R* (24) with 10,000 bootstrap iterations. The ROC curves are presented in **Figure 2**.

AUC for the UGDS-GS-PL Gender dysphoria subscale was.97, indicating excellent classification performance. The optimal cutoff value was 31, 95% boot CI [31.0, 32.0]. At this threshold, the model accuracy was .93, 95% boot CI [.92, .94], sensitivity was.94, 95% boot CI [.92, .94], and specificity was .92, 95% boot CI [.89, .94]. The Puberty-related distress subscale showed more moderate classification ability: AUC = .81, 95% CI [.79, .83], with the optimal cutoff of 8 (95% boot CI [7.0, 8.0]), yielding a sensitivity of.86, 95% CI [.83, .87], a specificity of .61, 95% CI [.50, .64], and an overall classification accuracy of .78, 95% CI [.75, .79]. Gender affirmation subscale showed limited diagnostic utility, AUC = .62, 95% boot CI [.58, .63]. Although the optimal cutoff score of 3.5, 95% CI [3.0, 12.0], maximized sensitivity (1.00, 95% boot CI [.79, 1.00]), it did so at the expense of extremely low specificity of .01 95% CI [.00, .08], resulting in unsatisfactory an overall accuracy of.67, 95% boot CI [.64, .67]. Such results indicate that this subscale does not meaningfully distinguish between cisgender and transgender/nonbinary respondents.

For comparison, the GIDYQ-AA-PL showed classification performance comparable to that of the UGDS-GS Gender Dysphoria subscale, with an area under the curve (AUC) of.98. However, using the cut-off point proposed by Niemiec et al. (8) performed worse than UGDS-GS-PL, with a sensitivity of.69, a specificity of.99, and an overall accuracy of.79. We therefore recalculated the optimal cutoff score in our sample and we found it was much lower than original (1.94, 95% CI [1.79, 2.06]), yielding a sensitivity of.97, 95% CI [.95,.98], a specificity of.87, 95% CI [.82,.89], and an overall accuracy of.93, 95% CI [.92,.94].

In sum, we evaluated whether the UGDS-GS-PL subscales could serve as effective screening tools for distinguishing transgender and nonbinary individuals from cisgender participants, and we compared them with those of the commonly used GIDYQ-AA-PL. The Gender dysphoria subscale showed excellent classification ability, Puberty-related distress performed moderately, and Gender affirmation showed poor diagnostic utility. When we applied the cutoff proposed by Niemiec et al. (8) to the GIDYQ-AA-PL, it performed poorly, yielding substantially worse classification results than our scale. This revised cutoff confirmed that the GIDYQ-AA-PL performs comparably to the UGDS-GS-PL Gender dysphoria subscale when optimally calibrated.

## Study 2

Having established the structure and validity for the Polish version of the UGDS-GS scale, we aimed to examine its reliability by testing its stability over time. We tested a group of Polish respondents twice over a period of three weeks. We expected good test-retest reliability for the dimensions of our UGDS-GS-PL scale. This study’s hypotheses, design, sample size, exclusions, and analyses were preregistered at https://aspredicted.org/7fxj-4vz2.pdf. As our initial analyses demonstrated a three-factor structure for the scale, we followed this structure in the reliability analyses (which deviated from preregistration). Additionally, we conducted non-preregistered CFA on the T1 data.

### Material and method

A total of 245 Polish participants (age: *M* = 27.71; *SD* = 7.50; 35 cisgender heterosexual, 139 cisgender non-heterosexual, 54 non-binary, and 17 binary transgender) from Prolific Academic took part in an online study at T1. Seven of them failed to provide correct answers to attention checks (same as in Study 1) and were excluded from the second part of the survey. The remaining 238 participants were invited to the second part of the survey in three weeks, and 160 completed it at T2 (age: *M* = 28.69; *SD* = 8.37; 23 cisgender heterosexual, 89 cisgender non-heterosexual, 33 non-binary, and 15 binary transgender).

Data were collected on Prolific at two time points (T1 and T2, separated by 3 weeks). At T1, after informed consent, participants provided demographics and completed the UGDS-GS-PL; at T2, they completed only the UGDS-GS-PL. Participants received £0.55 for participation in T1 and £0.60 for participation in T2.

### Results

#### Confirmatory factor analysis

We first conducted three CFAs on the T1 data to provide additional information on the structure of the UGDS-G-PL scale in a different sample. We began with an original two-factor structure as demonstrated by McGuire et al. (2020), then transitioned to a unidimensional structure. Finally, we tested a three-factorial structure, identifying Gender dysphoria, Puberty-related distress, and Gender affirmation as three separate but correlated factors. The two-factorial model estimated with the MLM method demonstrated poor fit, χ^2^/*df* = 3.47, RMSEA = .101, 90% CI [.092, .110], SRMR = .150, CFI = .88, TLI = 0.86, same as the uni-dimensional model, χ^2^/*df* = 3.46, RMSEA = .100, 90% CI [.091, .109], SRMR = .083, CFI = .88, TLI = 0.86. However, the novel three-factor model demonstrated good values for all fit indices, χ^2^/df = 2.42, RMSEA = .076, 90% CI [.067, .086], SRMR = .077, CFI = .93, TLI = 0.92. In this model, all factor loadings were significant, but two did not reach the .40 threshold (**Table 2**).

#### Internal consistency

Gender dysphoria again demonstrated excellent internal consistency (ω = .94, AVE = .62), whereas Puberty-related distress demonstrated acceptable consistency (ω = .78, AVE = .58). In contrast, Gender affirmation demonstrated just acceptable internal consistency (ω = .70, AVE = .46). This pattern of results matches the pattern from Study 1.

#### Stability over time

Gender dysphoria demonstrated excellent test-retest reliability (Pearson *r* = .92, *p* < .001), similar to that of Puberty-related distress (Pearson *r* = .85, *p* < .001). However, Gender affirmation demonstrated only moderate test-retest reliability (Pearson *r* = .58, p < .001), though suggesting this dimension may be somewhat subject to measurement error or state-dependent compared to the other subscales.

## General discussion

This research validates the Polish version of the Utrecht Gender Dysphoria Scale - Gender Spectrum (UGDS-GS-PL), addressing a critical gap in assessment tools for gender dysphoria that include nonbinary and gender-diverse individuals. Across two preregistered studies with *N* = 1265 participants, we identified an empirically derived factor structure that aligns with and extends the original conceptualization of the scale and established the instrument’s psychometric properties by demonstrating its convergent validity and measurement stability.

Our findings reveal an important departure from the original structures of the scale. Whereas the original version UGDS-GS identified Gender dysphoria and Gender affirmation as two primary dimensions (11, 12), the two-factor structure of the UGDS-GS scale did not hold in its adaptations to other languages. The results of exploratory factor analyses conducted on Chinese and Thai samples yielded different factor solutions: three in the Chinese adaptation (15), and four in the Thai version (14). In the Turkish adaptation, the Gender affirmation dimension was excluded, resulting in a unidimensional structure (16). Both the Thai and Chinese adaptations also reported a total score, which might imply an assumption of the existence of the higher-order general factor. While these results are inconsistent, they may suggest cultural differences in transgender experience affecting the scale’s factor structure. Thus, future adaptations of the UGDS-GS should not assume that the original factor structure will automatically replicate but should instead consider empirically deriving the structure most appropriate for the cultural context.

Consequently, our exploratory and confirmatory factor analyses consistently supported a three-factor solution comprising Gender dysphoria, Puberty-related distress, and Gender affirmation. This structure emerged robustly in two different samples, suggesting it represents a meaningful refinement rather than a cultural or methodological artifact. The identification of a three-factor structure in the Polish adaptation raises important questions about the cross-cultural generalizability of gender dysphoria constructs. This divergence could reflect several possibilities: (1) genuine cultural differences in how gender dysphoria is experienced or conceptualized, (2) linguistic differences in how gender-related constructs are expressed and interpreted in Polish versus English and Chinese/Taiwanese, or (3) methodological differences in sample composition or analytical approaches.

The emergence of Puberty-related distress as a distinct factor has important theoretical implications. Although strongly correlated with the broader Gender dysphoria dimension, Puberty-related distress appears to capture a specific developmental experience that warrants separate consideration. This finding aligns with clinical observations that pubertal development often represents a particularly distressing period for transgender and nonbinary individuals, as secondary sex characteristics emerge that conflict with one’s gender identity (29). The distinction between general gender dysphoria and puberty-specific distress may have practical utility in clinical settings, particularly when considering the timing and nature of gender-affirming interventions. For instance, individuals seeking puberty suppression or those experiencing distress specifically related to pubertal changes may show elevated scores on this dimension while reporting lower levels of general gender dysphoria.

The UGDS-GS-PL offers substantial advantages over the currently available GIDYQ-AA-PL (8). Most critically, the UGDS-GS-PL was designed from the outset to be accessible and appropriate for individuals across the gender spectrum, including nonbinary, genderqueer, and genderfluid individuals. The GIDYQ-AA-PL, by contrast, uses binary language that forces respondents to evaluate their experiences as “feeling like a woman” or “feeling like a man.” This binary framework creates significant difficulties for nonbinary individuals, who may find the items difficult or impossible to answer authentically, potentially leading to invalid responses or measurement error. In addition, the cut-off points proposed by Niemiec et al. (2023) did not perform well when applied to our data. Although the GIDYQ-AA-PL showed overall classification performance comparable to that of the UGDS-GS-PL Gender dysphoria subscale, the recommended cut-off produced substantially weaker results than the UGDS-GS-PL. This pattern suggests that the original cut-off point may have been calibrated to a sample whose composition differed markedly from ours. Specifically, in the sample used by Niemiec et al. (8), people with gender congruence constituted the majority (73.7%). Such an overrepresentation of gender-congruent participants likely inflated specificity at the expense of sensitivity, leading to a threshold that performs poorly in more gender-diverse samples. In contrast, our analyses were conducted on a substantially more heterogeneous group of participants, providing a more appropriate context for evaluating screening accuracy in gender-diverse populations.

Our careful translation process prioritized the use of gender-neutral grammatical forms and pronouns in Polish, a language with extensive grammatical gender. This linguistic achievement ensures that individuals of any gender identity can complete the scale without experiencing misgendering during the assessment process. Given that misgendering has been demonstrated to act as a significant minority stressor contributing to psychological distress (30, 31), the gender-neutral design of the UGDS-GS-PL represents not only a methodological improvement but also an ethical advancement that minimizes potential harm to respondents.

Furthermore, the UGDS-GS-PL’s inclusion of a Gender affirmation dimension provides information about positive aspects of gender identity that the GIDYQ-AA-PL does not capture. While the GIDYQ-AA-PL focuses primarily on distress and dissatisfaction, the UGDS-GS-PL acknowledges that gender identity encompasses both challenges and positive experiences. This more comprehensive assessment approach aligns with contemporary models of transgender health that emphasize resilience, affirmation, and well-being rather than focusing exclusively on pathology and distress. The readability of the UGDS-GS-PL is also superior, with a Gunning-Fog index indicating comprehensibility at the primary education level. This accessibility ensures that the scale can be used with diverse populations, including individuals with varying levels of education, and reduces the likelihood of measurement error due to item miscomprehension.

The UGDS-GS-PL demonstrated strong convergent validity, with the Gender dysphoria dimension showing excellent correlation with the established GIDYQ-AA-PL. This substantial relationship provides confidence that the scale measures the intended construct while offering advantages in terms of gender-neutral language and accessibility for nonbinary individuals. Importantly, the moderate correlations between Gender dysphoria dimension and measures of general psychopathology (anxiety and depression) suggest that gender dysphoria, while associated with psychological distress, represents a distinct construct rather than merely reflecting general mental health difficulties. This finding supports the conceptualization of gender dysphoria as a specific experience related to gender identity rather than a manifestation of broader psychopathology.

The Puberty-related distress dimension showed similar patterns of convergent validity, with strong associations with the GIDYQ-AA-PL and moderate correlations with anxiety and depression. These relationships suggest that puberty-related distress, while relatively distinct from general gender dysphoria, is similarly associated with psychological difficulties and represents a clinically meaningful dimension of gender-related distress.

The Gender affirmation dimension, however, demonstrated suboptimal psychometric properties, warranting cautious interpretation and substantial concern. While it showed a weak positive correlation with the GIDYQ-AA-PL and weak negative correlations with anxiety and depression—patterns that superficially align with theoretical expectations—the overall evidence raises serious questions about the adequacy of this dimension. The internal consistency fell below conventional standards, with omega coefficients and average variance explained values suggesting inadequate item cohesion. Most troublingly, the test-retest reliability was only moderate, substantially lower than the excellent stability demonstrated by the distress-related dimensions. This instability may be interpreted as measurement error or conceptual ambiguity in how the construct is operationalized. Moreover, existing literature suggests that gender affirmation experiences may fluctuate over time depending on individual experiences of affirmation (32) and participants’ psychological state (33) indicating that this dimension may capture a state rather than a stable construct.

The weak and theoretically ambiguous pattern of correlations with other measures further undermines confidence in what this dimension actually captures. While one might expect stronger associations with positive mental health indicators or other measures of gender identity satisfaction, the observed relationships were modest at best. However, the convergent validity evidence might be insufficient, especially as we did not include additional measures of positive gender identity experiences that could have clarified what Gender affirmation represents.

Additionally, the relatively weak and unstable performance of this dimension may be partly attributable to cross-cultural factors. Gender affirmation is inherently relational and socially embedded: it depends not only on internal identity processes but also on social recognition, validation, and the broader cultural scripts surrounding gender expression and transition. Because norms regarding gender roles, the visibility of transgender identities, access to gender-affirming resources, and levels of societal acceptance vary across cultural contexts, the lived meaning of “affirmation” may differ as well. In some settings, affirmation may be closely tied to medical or legal transition processes; in others, it may be primarily interpersonal, symbolic, or community-based. As a result, items intended to capture a unified construct may function differently across cultural contexts, potentially leading to reduced internal consistency and a less coherent factor structure. Future cross-cultural measurement invariance studies would be necessary to determine whether the observed weaknesses reflect methodological limitations of the current operationalization or genuine contextual differences in how gender affirmation is experienced and conceptualized.

Users should therefore exercise considerable caution when interpreting Gender affirmation scores. While the conceptual importance of measuring positive aspects of experiencing gender is clear and theoretically compelling, the current operationalization appears problematic and requires substantial refinement before it can be confidently used in clinical or research contexts. This dimension should be considered preliminary and experimental rather than established. Future work must focus on developing additional items, clarifying the theoretical definition of gender affirmation, establishing stronger validity evidence by including related positive gender identity measures, and determining whether the construct’s apparent instability reflects genuine state-dependence or measurement inadequacy. Until these improvements are made, we recommend that researchers and clinicians prioritize the two distress-related dimensions, which demonstrate robust psychometric properties, and treat Gender affirmation scores with skepticism.

The UGDS-GS-PL demonstrated excellent internal consistency for Gender dysphoria and acceptable to good consistency for Puberty-related distress across both studies. These dimensions showed item cohesion and discrimination indices that indicate the items are measuring unified constructs and differentiating well between individuals with varying levels of distress. Test-retest reliability over three weeks was excellent for Gender dysphoria and Puberty-related distress, indicating that these dimensions capture relatively stable individual differences. These results are comparable to those reported in the Turkish adaptation, where the ICC coefficient was 0.884, suggesting similarly high stability over time. This stability is essential for both cross-sectional research comparing groups and longitudinal research tracking changes over time. The robust test-retest correlations provide confidence that these dimensions are measuring enduring characteristics rather than transient states, making them suitable for clinical assessment and research applications.

### Limitations

Several limitations should be considered when interpreting these findings. First, while our sample was large and diverse in terms of gender identity, it was primarily recruited through online platforms and LGBTQ+ social media groups, which may limit generalizability to individuals who are less connected to LGBTQ+ communities or who have limited internet access. The recruitment strategy may have resulted in a sample that is more open about their gender identity and more knowledgeable about gender-related terminology than the broader population of gender-diverse individuals in Poland.

Second, the Gender affirmation dimension demonstrated inadequate reliability and unclear validity, requiring substantial refinement and additional validation before it can be confidently interpreted. The moderate test-retest reliability, suboptimal internal consistency, and weak pattern of correlations with other constructs raise fundamental questions about what this dimension measures and whether it captures a stable, meaningful aspect of gender identity experience. This represents a significant limitation of the current version of the scale and should be a priority for future research. Until these psychometric issues are addressed, the Gender affirmation subscale should be considered experimental and used only with appropriate caution and caveats.

Third, we did not include additional measures of positive gender identity experiences beyond the Gender affirmation subscale, limiting our ability to fully assess the convergent validity of this dimension. This omission is particularly problematic given the Gender affirmation subscale’s psychometric weaknesses. Future research would benefit from including measures of gender identity pride, comfort with one’s gender expression, satisfaction with gender-affirming interventions, and other positive aspects of gender identity to more comprehensively evaluate whether the Gender affirmation dimension captures what it purports to measure and whether refinements improve its validity.

Fourth, the UGDS-GS-PL was validated in Polish, and the three-factor structure may not generalize to other linguistic or cultural contexts. The original two-factor structure identified by McGuire et al. (11, 12) was based on English-speaking samples, and it remains unclear whether the three-factor structure represents a universal improvement or a culturally specific finding. Cross-cultural validation studies are needed to determine the generalizability of our factor structure and to understand whether puberty-related distress emerges as a distinct factor in other cultural and linguistic contexts. Moreover, the Polish language’s grammatical gender system and cultural context surrounding gender may influence how individuals interpret and respond to items on the scale. While our translation process carefully maintained gender-neutral language where possible, the cultural meanings and salience of gender-related concepts may differ between different languages and cultures. Future research should examine whether the three-factor structure emerges in other linguistic and cultural contexts to determine whether it represents a universal refinement of the scale or a culturally specific manifestation.

Fifth, while the high test-retest reliability over three weeks indicates stability in the absence of intervention, it remains unknown whether the scale can detect meaningful changes in gender dysphoria following medical or social transition. Longitudinal research examining the scale’s responsiveness to intervention is an important direction for future research, particularly since a key clinical application would be monitoring treatment outcomes.

A further limitation concerns the lack of measurement invariance testing across gender identity groups. Although the total sample size was sufficient for single-group CFA, the subgroup sizes—particularly for cisgender heterosexual and binary transgender participants—were relatively small for conducting robust multi-group CFA with sequential invariance constraints. As a result, we were unable to determine whether the scale functions equivalently across groups. Consequently, observed between-group differences should be interpreted with caution, as they may reflect not only true differences in gender dysphoria but also potential differences in item functioning. Future research with larger and more balanced subgroup samples should formally test measurement invariance across gender identity categories.

Finally, another limitation concerns the restricted scope of sociodemographic data collected in this study. We did not obtain detailed information regarding participants’ education level, place of residence, or socioeconomic status. This decision was made intentionally to minimize participant burden and protect the perceived anonymity of a potentially vulnerable population (non-cisgender and non-heterosexual individuals), for whom disclosure of personal background characteristics may be experienced as sensitive. However, the absence of these variables limits our ability to assess the representativeness of the sample and to evaluate the generalizability of the findings across different sociodemographic groups. Future research should incorporate more comprehensive background measures, while carefully balancing the need for contextual data with ethical considerations related to participant safety and confidentiality.

Despite these limitations, the UGDS-GS-PL represents a significant advancement in the assessment of gender dysphoria in Poland, particularly concerning its distress-related dimensions. By offering a measure that is inclusive, accessible, and—for Gender dysphoria and Puberty-related distress—psychometrically sound, this research supports both clinical practice and research aimed at understanding and supporting the well-being of transgender and gender-diverse individuals in Poland. The scale provides a valuable alternative to existing binary-focused instruments and demonstrates that gender-neutral assessment of gender dysphoria is both feasible and psychometrically viable in the Polish language.

## Conclusions

The UGDS-GS-PL is a psychometrically sound tool for assessing gender dysphoria among Polish transgender, nonbinary, and cisgender individuals, particularly through its two reliable distress-related dimensions: Gender dysphoria and Puberty-related distress. Its three-factor structure both confirms the multidimensional nature of gender-related distress and offers a refinement of the original scale. By using gender-neutral language and avoiding binary assumptions, the UGDS-GS-PL fills a critical gap in Polish assessment and provides clinicians and researchers with an inclusive, nuanced measure of gender-related experiences. The scale minimizes risks of misgendering and represents an ethical improvement over existing tools. The Gender affirmation dimension, however, shows insufficient reliability and validity and should be interpreted with caution, if at all. At present, it should be treated as an experimental component, while clinical and research use should focus on the two distress-related dimensions supported by strong psychometric evidence.

Importantly, the UGDS-GS-PL is intended to support—not limit—access to gender-affirming care. Scores should never be used to gatekeep interventions. The scale’s value lies in facilitating communication, identifying sources of distress, and tracking changes over time. Clinically, it may assist in identifying puberty-related distress, monitoring dysphoria across transition processes, distinguishing types of distress to guide interventions, and supporting therapeutic dialogue. All clinical decisions should remain grounded in individuals’ self-reported experiences, needs, and transition goals.

## Data Availability

The datasets presented in this study can be found in online repositories. The names of the repository/repositories and accession number(s) can be found in the article/supplementary material.
